# A Rare Case of Immune Thrombocytopenia With Intracranial Hemorrhage Secondary to Cavernous Malformation

**DOI:** 10.7759/cureus.64705

**Published:** 2024-07-17

**Authors:** Devika Jadhav, Pranavi Mokkarala, Sampada Tambolkar, Pooja Sadhu

**Affiliations:** 1 Pediatrics, Dr. D. Y. Patil Medical College, Hospital & Research Centre, Dr. D. Y. Patil Vidyapeeth (Deemed to be University), Pune, IND

**Keywords:** immune thombocytopenia, cavernous malformation, arteriovenous malformation, seizures, intracranial hemorrhage, bleeding in children

## Abstract

Immune thrombocytopenia (ITP) is an autoimmune disease resulting in a fall in platelet count, causing ecchymoses and bleeding manifestations. The most prevalent acquired bleeding disorder in children is ITP. Intracranial hemorrhage (ICH) is a rare but most devastating complication of ITP which can cause neurological sequelae. We report the case of a four-year-old male child who presented with a history of seizures, headache, multiple ecchymoses, and bruising. Blood counts and bone marrow examination were suggestive of ITP. Magnetic resonance imaging (MRI) of the brain showed ICH with multiple cerebral cavernous malformations. ICH as the first presentation of ITP is extremely rare. ICH in a case of ITP secondary to arteriovenous malformation has been scarcely reported, establishing the rarity of the currently presented case. Morbidity and mortality of ICH occurring as a consequence of ITP can be reduced by recognizing the symptoms, diagnosing promptly, and treating aggressively.

## Introduction

The hallmarks of immune thrombocytopenia (ITP) are low platelet counts, purpura, and hemorrhagic episodes caused by antiplatelet autoantibodies [1]. The incidence of acute ITP in children is 1.1 per 105 children/year and that of chronic ITP is 0.46 per 105 children/year [2]. In a prospective population-based study from the five Nordic countries between 1998 and 2000, the annual incidence of ITP was 4.8 per 100,000 children younger than 15 years of age. While ITP can manifest in children at any age, the condition is more common in those between the ages of two and five years [3]. There is a minor male-to-female predominance, particularly in infants [4]. By contrast, there is a female predominance of ITP in adolescents and younger adults [5].

Children with ITP usually present with purple bruise-like patches and mucosal bleeds. Rarely do patients present with neurological signs of intracranial hemorrhage (ICH). A survey of ICH in ITP reveals that head trauma preceded ICH in 33% of patients. Patients with ICH tended to have more frequent bleeding than just skin petechiae and ecchymoses. The ICH was lethal in 25% [6].

According to a literature review done in 2015, out of the 114 ICH cases documented in children with ITP, 26%, i.e. 30, were found to have had head trauma. Thirteen percent of these 30 subjects who had an ICH associated with a head injury suffered severe neurological consequences, and 23 died as a result of the ICH. Twenty-seven percent of patients were aged three years or younger at the time of presentation [7].

## Case presentation

A four-year-old male child was brought to a local hospital with an alleged history of a fall on the ground from a height of about two feet, followed by the development of tonic-clonic movements of all four limbs associated with uprolling of eyes which lasted for 10 minutes. Parents also noticed swelling and bruising on the forehead. The seizure was aborted by using intranasal midazolam spray followed by a loading dose of intravenous (IV) levetiracetam. The child underwent a CT brain, which showed an intraparenchymal hemorrhage in the right internal capsule. He was brought to our institution for further management two days later.

On clinical examination, the child was hemodynamically stable. The child did not have pallor, lymphadenopathy, organomegaly, or bone pain. Neurological examination was normal. There were multiple bruises and ecchymoses all over his body. Blood investigations showed normal hemoglobin and white cell count with severe thrombocytopenia (platelets = 14,000/µL). Prothrombin levels were normal (Table 1). Peripheral smear was suggestive of marked thrombocytopenia.

**Table 1:** Blood investigations on admission

Complete Blood Picture - Parameters		Values
Hemoglobin		12.10 g/dL
Total leucocyte count		7,900/μL
Differential leucocyte count	Lymphocytes	54%
	Neutrophils	33%
	Eosinophils	3%
	Basophils	0%
	Monocytes	10%
Platelet count		14,000/μL
Red blood cell count		4.85 million/μL
Hematocrit		35.40%
C-reactive protein		3.20 mg/L
Erythrocyte sedimentation rate		25
Prothrombin time	Patient value	12.00 seconds
	Control value	11.80 seconds
International normalized ratio		1.02
Activated partial thromboplastin time	Patient value	25.10 seconds
	Control value	25.23 seconds

The repeat platelet count was 9,000/µL. Bone marrow cytology showed normal bony trabeculae, with intertrabecular spaces showing hemorrhage. A section from the bone marrow clot showed hypocellular marrow particles (70% marrow cellularity), and cells of erythroid and myeloid series showed progressive maturation with mildly increased megakaryocytes, suggestive of ITP.

Random donor platelets (RDPs) were transfused and the child was started on IV dexamethasone, which was later changed to IV methylprednisolone.

Koch’s work-up was done to rule out tuberculosis as the child's mother had a history of tuberculosis and neurotuberculosis was considered one of the differential diagnoses - no abnormalities were detected. Abdominal ultrasonography showed borderline splenomegaly.

Despite multiple RDP transfusions and continuing IV steroids over the next few days, the platelet count decreased to 4,000/µL on the fifth day of admission (Table 2).

**Table 2:** Serial blood counts over the next few days showing a downward trend of platelets

Blood Parameters	Day 2	Day 3	Day 4	Day 5
Hemoglobin (g/dL)	11.50	9.70	10.40	9.20
Total leucocyte count (/μL)	13,500	6,200	3,870	3,640
Platelet count (/μL)	9,000	8,000	7,000	4,000
Hematocrit (%)	35.90	28.60	31.40	27.90

**Figure 1:**
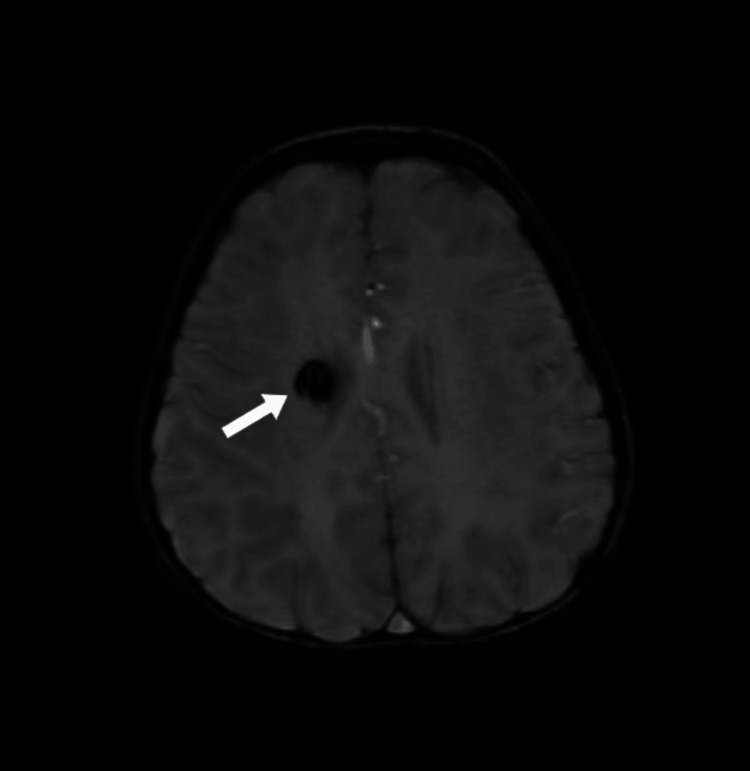
The white arrow points to a cerebral cavernous venous malformation near the right corona radiata

**Figure 2:**
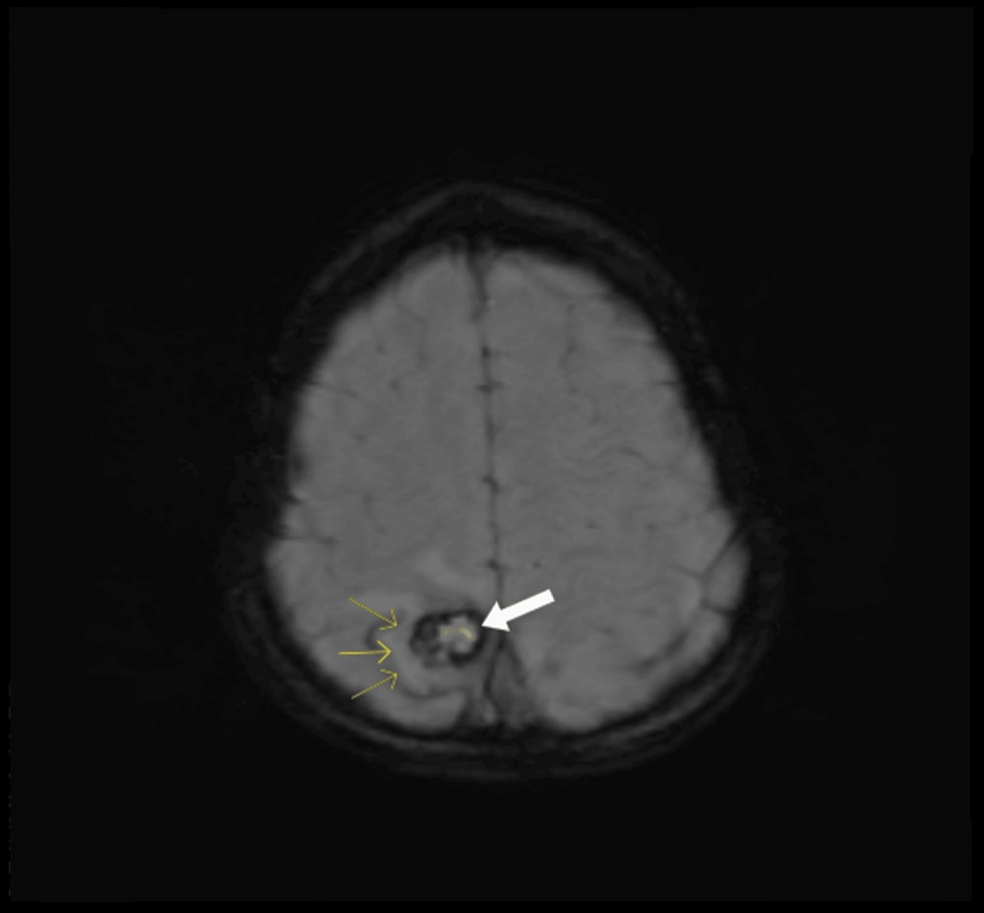
The white arrow points to a cerebral cavernous venous malformation in the right high parietal region, and the yellow arrows point to the perilesional edema

Magnetic resonance imaging (MRI) brain (plain + contrast) was reported as follows: Three well-defined lesions with susceptibility-weighted imaging (SWI) blooming were seen in the right corona radiata (Figure 1), right high parietal (Figure 2), and the left basifrontal regions with no enhancement. Marked perilesional edema was seen in the right high parietal region (Figure 2). The findings are suggestive of cerebral cavernous venous malformations (CCMs) with multiple cavernomas of different stages.

The patient was then given IV immunoglobulin (IVIG) and one dose of injection romiplostim, following which his platelet counts started to rise (Table 3).

**Table 3:** Serial blood counts after administration of intravenous immunoglobulin and injection romiplostim

Blood Parameters	Day 6	Day 7
Hemoglobin (g/dL)	9.00	10.40
Total leucocyte count (/μL)	4,520	7,400
Platelet count (/μL)	37,000	184,000
Hematocrit (%)	27.00	32.10

The child was discharged on a platelet count of 184,000/µL. He has been on regular follow-up and continues to have a normal platelet count. No neurological sequelae have been observed.

## Discussion

The pathophysiology of ITP involves autoantibodies that mark platelets to be destroyed by macrophages from the reticuloendothelial system, commonly from the spleen. The bone marrow makes up for platelet loss by accelerating platelet production. ITP typically manifests in healthy children within a few weeks of contracting a viral infection.

Other causes may be carcinomas (e.g., adenocarcinoma and lymphoma), autoimmune diseases, common variable immunodeficiency, and certain drugs [1].

Patients with ITP alone were observed to have a mortality of 20% [8]. The recovery rate of ITP is 77% in infants, 68.6% in older children, and 32-53% in adolescents [9]. According to a case-control study done in 2009, the incidence of ICH in children with ITP was estimated to be 0.19-0.78% [6]. Albeit uncommon, it is the most dreadful complication of ITP. The combination of ICH and ITP doubles the mortality [9]. The features that put ITP patients at risk of developing ICH include platelet counts below 10,000-20,000/µL, head trauma, usage of nonsteroidal anti-inflammatory drugs, systemic lupus erythematosus-associated vasculitis, and, rarely, cerebral arteriovenous malformations (AVMs) [6]. Children with ICH present with headache, vomiting, altered sensorium, convulsions, and focal neurological deficits.

CCMs are clusters of unusual, hyalinized capillaries with no brain tissue in between. These lesions have low pressure and slow flow, resulting in a significantly lower average risk of rupture than certain other vascular malformations, like AVMs. Although usually found incidentally, cavernomas can also be discovered during the assessment of headaches, seizures, focal neurologic deficits, or symptomatic hemorrhage. The gross pathological appearance of CCMs is often described as a "mulberry." The incidence of cavernomas ranges from 0.4% to 0.8% in the population, occurring more commonly in the fourth decade [10]. However, they are linked to a greater risk of bleeding in children compared to adults [11].

Very few cases have been documented describing cavernomas in ITP. ICH in a case of ITP secondary to AVM has been reported in only two cases according to a literature review done over 46 years [12]. The child reported in this case had multiple risk factors for ICH like ITP and CCMs, which caused it to occur even before he could be diagnosed with ITP.

Management of ITP includes multiple options. Steroids promote the integrity of blood vessels, reduce the synthesis of antiplatelet autoantibodies, and minimize the splenic destruction of antibody-coated platelets [13]. The thrombopoietin (TPO) analog romiplostim works by binding to the TPO receptor and triggering the formation of bone marrow megakaryocyte colony-forming cells, which results in increased platelet synthesis [14]. IVIG acts by blocking the Fc receptor, which reduces the destruction of sensitized platelets in the spleen [15]. When conservative management is not sufficient, surgical options like craniotomy and splenectomy are considered.

## Conclusions

ICH as the first presentation of ITP is extremely rare but potentially fatal. The possibility of AVM should be considered in a child presenting with ICH with ITP. An early MRI of the brain will help establish the diagnosis and start treatment promptly. Neurological symptoms in childhood ITP should be recognized early, promptly assessed with MRI, and managed aggressively, to prevent morbidity and mortality associated with ICH in a patient of ITP.
